# Complete mitochondrial genome sequence of the northern red-shouldered macaw (*Diopsittaca nobilis*)

**DOI:** 10.1080/23802359.2020.1847610

**Published:** 2021-01-13

**Authors:** Da-wei Liu, Jian He, Chun-ping Xie

**Affiliations:** a Nanjing Forest Police College, Nanjing, China;; b Forensic Identification Center for Forest Police of the State Forest Bureau, Nanjing, China;; c Key Laboratory of State Forest and Grassland Administration Wildlife Evidence Technology, Nanjing, China

**Keywords:** Red-shouldered macaw (*Diopsittaca nobilis*), mitochondrial genome, phylogenetic analysis

## Abstract

The northern red-shouldered macaw, *Diopsittaca nobilis*, is listed in the Convention on International Trade in Endangered Species of Wild Fauna and Flora Appendix II. Here, we report for the first time the complete mitochondrial genome of *D. nobilis.* This new sequence is 16,992 base pairs (bp) in length and includes 13 protein-coding genes, 22 transfer RNAs, two ribosomal RNAs, and a single non-coding control region. The overall nucleotide composition of this sequence consists of 30.40% A, 14.00% G, 23.60% T, and 32.00% C. The phylogenetic relationships suggested that the mitogenome of *D. nobilis* is close to that of three other macaw species. Our results provide useful mitogenomic information to support further studies on the phylogeny and taxonomy of Psittaciformes.

The northern red-shouldered macaw (*Diopsittaca nobilis*) belongs to the family Psittacidae (Aves, Psittaciformes). The geographic range of this species spans across Brazil, French Guiana, Guyana, Suriname, and Venezuela (BirdLife International 2020), where it inhabits savanna formations and forest borders (Collar 1997). Its population is considered to be stable, as there is no evidence of any declines or substantial threats. Therefore, the extinction risk of this species has been evaluated as of ‘Least Concern’ by the International Union for Conservation of Nature (IUCN 2020). Nonetheless, *D. nobilis* is listed in the Convention on International Trade in Endangered Species of Wild Fauna and Flora Appendix II, owing to its association with wild animal trafficking. In animals, the mitochondrial genome (mtDNA) is a closed double-stranded circular molecule (Boore 1999). Mitogenome data have been widely used for phylogenetic analysis, owing to several advantages, including rapid evolution, maternal inheritance, and simple structure (Harrison 1989). However, because the analysis of single or incomplete gene sequences can lead to incorrect inferences, entire mitogenome sequences are indispensable for elucidating phylogenetic relationships (Russo et al. 1996; Urantowka et al. 2017). Therefore, the complete mitogenome of *D. nobilis* was determined.

A feather sample of *D. nobilis* was collected in the Hongshan Zoo, Nanjing, Jiangsu Province, China (32°09′N, 118°08′E). After sampling, the specimen (A-2019004) was stored in the Key Laboratory of Wildlife Evidence Technology State Forest and Grassland Administration, Nanjing, Jiangsu Province, China. Genomic DNA was isolated using a Universal Genomic DNA Extraction Kit (Takara, Beijing, China) in a final elution volume of 60 μL. PCR primers were designed based on the alignment of the complete mtDNA sequences of *Ara ararauna* (GenBank accession no. KF010315) and *A. glaucogularis* (GenBank accession no. JQ782215). Finally, PCR products were subjected to Sanger sequencing, and all the fragments were sequenced in both directions. The Sanger sequences were assembled using the SeqMan program of Lasergene 7.1 (DNAStar, Inc., Madison, WI).

The complete mitogenome of *D. nobilis* was determined to be 16,992 base pairs (bp) in length, and has been deposited in GenBank under the accession number MT740319. The mitogenome of this species is a circular structure, which is typical in birds (Liu et al. 2019; Sun et al. 2020), and includes 13 protein-coding genes, 22 transfer RNAs, two ribosomal RNAs, and a single non-coding control region. The overall nucleotide composition of the sequence is as follows: A = 5165 bp (30.40%); G = 2379 bp (14.00%); T = 4010 bp (23.60%); and C = 5438 bp (32.00%).

Furthermore, the phylogenetic analysis was constructed utilizing maximum likelihood (ML) method. According to the Akaike information criterion (AIC), the best-fit substitution model for ML method was selected by ModelFinder and was determined as GTR + F + I + G4. ML analyses were performed with IQ-TREE v 1.6.8 (Nguyen et al. 2015), with standard bootstrap for 1000 replicates. This analysis was based on the whole mitogenome sequences of *D. nobilis* and those of nine other species, namely *Primolius couloni*, *Ara severus*, *Orthopsittaca manilatus*, *Rhynchopsitta terrisi*, *Pyrrhura rupicola*, *Melopsittacus undulatus*, *Eclectus roratus*, *Probosciger aterrimus goliath*, and *Eolophus roseicapillus*. The outgroup was the complete mitochondrial genome of *Upupa epops*. Notably, our target species, *D. nobilis* and the clade (*O. manilatus* + (*P. couloni* + *A. severus*)) were clustered into one branch (Figure 1). Overall, the newly characterized *D. nobilis* mitogenome will provide useful genetic data to understand the evolutionary relationships among Psittaciformes species, and provide fundamental information for genetic conservation of this species.

**Figure 1.**
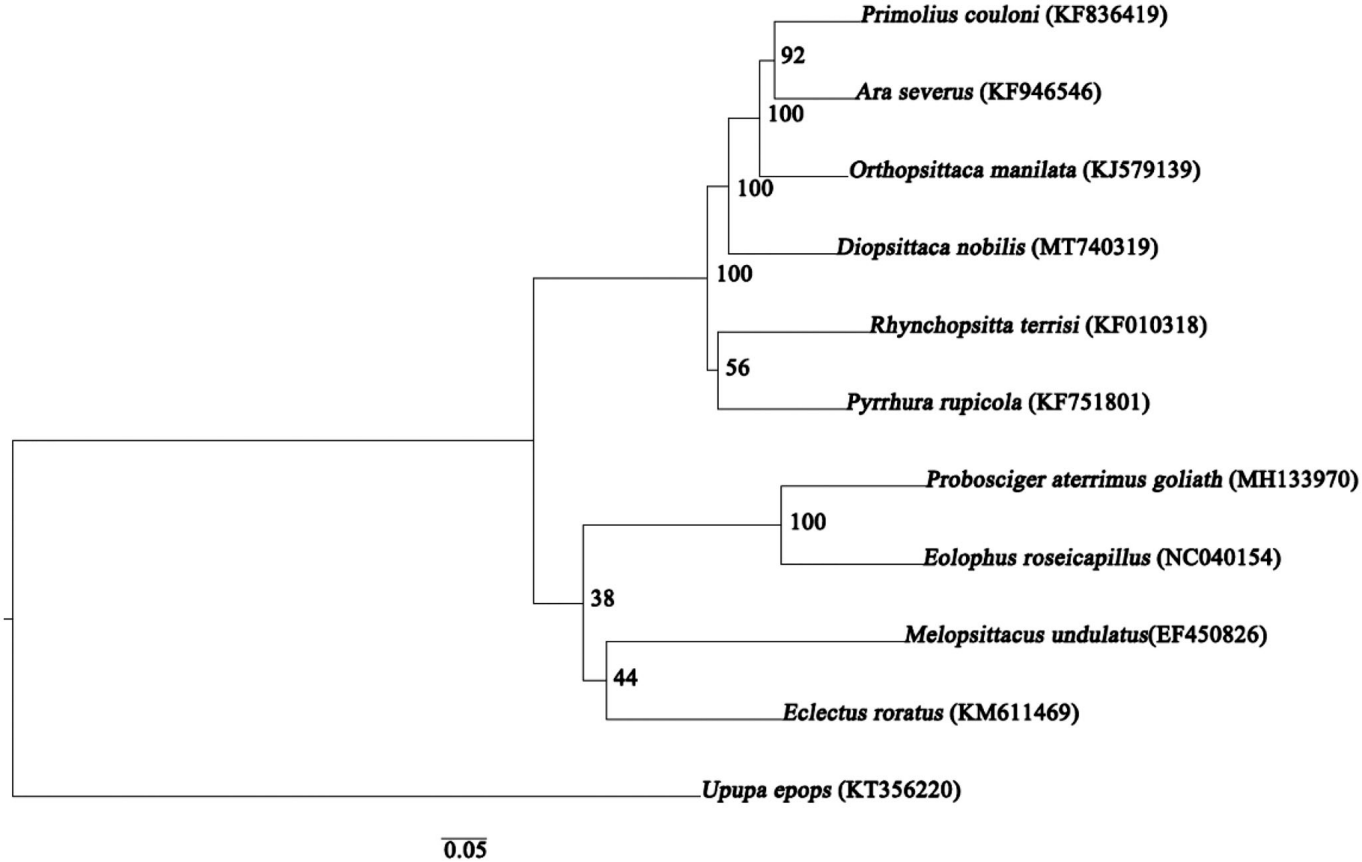
Maximum likelihood tree based on the complete mitogenomes of *Diopsittaca nobilis* (MT740319) with nine other avian species. Respective GenBank accession numbers are provided in parentheses. The bootstrap values are listed for each node.

## Data Availability

The data that support the findings of this study are openly available in GenBank at https://www.ncbi.nlm.nih.gov, reference number MT740319.
